# Preliminary exploration of amide proton transfer weighted imaging in differentiation between benign and malignant bone tumors

**DOI:** 10.3389/fonc.2024.1402628

**Published:** 2024-06-06

**Authors:** Ying Li, Liangjie Lin, Yong Zhang, Cuiping Ren, Wenhua Zhang, Jingliang Cheng

**Affiliations:** ^1^ Department of Magnetic Resonance Imaging, The First Affiliated Hospital of Zhengzhou University, Zhengzhou, China; ^2^ Clinical and Technical Support, Philips Healthcare, Beijing, China

**Keywords:** bone tumor, amide proton transfer weighted imaging, diffusion weighted imaging, differential diagnosis, magnetic resonance imaging

## Abstract

**Purpose:**

To explore the value of 3D amide proton transfer weighted imaging (APTWI) in the differential diagnosis between benign and malignant bone tumors, and to compare the diagnostic performance of APTWI with traditional diffusion-weighted imaging (DWI).

**Materials and methods:**

Patients with bone tumors located in the pelvis or lower limbs confirmed by puncture or surgical pathology were collected from January 2021 to July 2023 in the First Affiliated Hospital of Zhengzhou University. All patients underwent APTWI and DWI examinations. The magnetization transfer ratio with asymmetric analysis at the frequency offset of 3.5 ppm [MTRasym(3.5 ppm)] derived by APTWI and the apparent diffusion coefficient (ADC) derived by DWI for the tumors were measured. The Kolmogorou-Smirnou and Levene normality test was used to confirm the normal distribution of imaging parameters; and the independent sample t test was used to compare the differences in MTRasym(3.5 ppm) and ADC between benign and malignant bone tumors. In addition, the receiver operating characteristic (ROC) curve was used to evaluate the diagnostic performance of different imaging parameters in differentiation between benign and malignant bone tumors. P<0.05 means statistically significant.

**Results:**

Among 85 bone tumor patients, 33 were benign and 52 were malignant. The MTRasym(3.5 ppm) values of malignant bone tumors were significantly higher than those of benign tumors, while the ADC values were significantly lower in benign tumors. ROC analysis shows that MTRasym(3.5 ppm) and ADC values perform well in the differential diagnosis of benign and malignant bone tumors, with the area under the ROC curve (AUC) of 0.798 and 0.780, respectively. Combination of MTRasym(3.5 ppm) and ADC values can further improve the diagnostic performance with the AUC of 0.849 (sensitivity = 84.9% and specificity = 73.1%).

**Conclusion:**

MTRasym(3.5 ppm) of malignant bone tumors was significantly higher than that of benign bone tumors, reflecting the abnormal increase of protein synthesis in malignant tumors. APTWI combined with DWI can achieve a high diagnostic efficacy in differentiation between benign and malignant bone tumors.

## 1 Introduction

Bone tumors are associated with various types, low incidence rates, diverse manifestations and complex origins. The WHO has released the fifth edition criteria in 2020 for the classification of bone and soft tissue tumors, where the overall classification of bone tumors has been reduced from the original 12 categories to 8 categories, covering a total of 68 subtypes of diseases (1). As the gold standard for diagnosis of many other tumors, the pathological diagnosis of bone tumors has always been challenging (2), not only because of the complex classification of bone tumors and the lack of specific molecular markers, but also because its high dependence to tumor puncture sampling. It is currently agreed that the diagnosis of bone tumors requires a comprehensive evaluation of clinical, pathological and imaging information.

With the development of medical imaging techniques, the diagnosis of bone lesions is no longer limited to the traditional X-ray, and the role of CT and MRI has become increasingly important (3). In particular, with the widespread applications of MRI in the bone and joint system, it is now playing an important role in early detection of bone tumor lesions. However, conventional MRI mainly provides morphological information of tumors that has low specificity in differentiating between benign and malignant bone lesions. Diffusion weighted MRI (DWI) has now been widely used in tumor diagnosis in various parts of the body (4). It can help with the differentiation between benign and malignant bone tumors by providing insight into the cellularity of lesions, where malignant tumors have markedly increased cellularity which can lead to more restricted diffusion reflected by high DWI signal and a low apparent diffusion coefficient (ADC) value (5–7). Dynamic contrast-enhanced MRI (DCE-MRI) has also been widely explored in bone tumor evaluation based on lesion enhancement pattern, which is shown semi-quantitatively with evaluation of the time intensity curve and quantitatively with pharmacokinetic model determination (8, 9). While the use of DWI and/or DCE-MRI for bone tumor evaluation still requires familiarity with potential diagnostic pitfalls due to technical challenges and other confounding biochemical factors, such as the large amount of lipids contained in the bone marrow (3, 10).

Amide proton transfer weighted imaging (APTWI) is a type of chemical exchange saturation transfer (CEST) MRI technique. By detection of the chemical exchange between amide and water protons, it allows for non-invasive and quantitative evaluation of endogenous mobile proteins and peptides in living tissues without the use of an external contrast agents, thereby indirectly reflecting the metabolic status of the tissue (11, 12). APTWI has been applied to various tumor studies, including brain tumors, head and neck tumors, cervical cancer, endometrial cancer, prostate cancer, rectal cancer, and breast cancer, etc. obtaining a series of clinically valuable results in tumor identification, grading, treatment evaluation, and prognosis monitoring (13–15).

This study aims to explore the potential clinical value of three-dimensional (3D) APTWI in the differential diagnosis between benign and malignant bone tumors in comparison to DWI.

## 2 Materials and methods

### 2.1 General information of the research subjects

This is a prospective study, which has received approval from the Ethics Committee of the First Affiliated Hospital of Zhengzhou University. 112 patients, who were clinically suspected of having bone tumors in the pelvis and lower limbs, were collected from January 2021 to July 2023. All patients signed an informed consent form before the examination. The inclusion criteria were as follows: 1. patients with suspected of bone tumors; 2. comprehensive clinical data available; 3. no interventional procedures such as pathological biopsy, chemotherapy, radiotherapy, or interventional therapy prior to the MRI examination; and 4. patients without contraindications for MRI scan. The exclusion criteria were as follows: 1. with severe image artifacts or unclear display of lesions affecting diagnosis or data measurement; 2. without completed pathological results by puncture biopsy or surgery within a week following the MRI examination; 3. unable to be included into defined groups.

### 2.2 Magnetic resonance imaging

MRI scans were performed on a 3T MRI scanner (Ingenia CX, Philips Healthcare, Best, the Netherlands) equipped with an 18-channel phased array body coil. The patients were scanned in the supine position, with both upper limbs placed in front of the chest and both lower limbs as close together as possible. Sandbags were placed in appropriate positions to help improve the B0 field homogeneity. The MRI scans were performed with parameters as follows: (1) Coronal T_1_ weighted imaging (T_1_WI): TR = 500 ms, TE = 20 ms, slice thickness = 5 mm, field of view = 380 × 380 mm^2^, acquisition matrix = 472 × 445, number of excitation = 1, scan time = 1 min 07 s; (2) Coronal T_2_ weighted imaging (T_2_WI): TR = 4000 ms, TE = 130 ms, slice thickness = 5 mm, field of view = 380 × 380 mm^2^, acquisition matrix = 400×360, number of excitations = 2, scan time = 1 min 39 s; (3) Axial fat-suppressed T_2_WI: TR = 5200 ms, TE = 90 ms, slice thickness = 5 mm, field of view = 380 × 260 mm^2^, acquisition matrix = 306×160, number of excitation = 1, scan time = 1 min 44 s; (4) Axial DWI: TR = 5500 ms, TE = 83 ms, slice thickness = 5 mm, field of view = 380 × 260 mm^2^, acquisition matrix = 190×136, b values = 0 and 1000 s/mm^2^, number of excitations = 2, scan time = 1 min 45 s; (5) Axial APTWI: TR = 5400 ms, TE = 9.8 ms, slice thickness = 5 mm, field of view = 230 × 345 mm^2^, acquisition matrix = 92×114, number of excitation = 1, scan time = 4 min 30 s, saturation duration and power were 2 s and 2 μT, respectively; saturation frequency points were: ± 2.7, ± 3.5, ± 4.3 and -1540 (the reference signal) ppm.

### 2.3 Image analysis and post-processing

Image reconstruction of APTWI and DWI was automatically completed on the MRI host. APTWI was quantified by calculating the asymmetry of the traditional magnetization transfer effect at 3.5 ppm on both sides of the water signal [asymmetric magnetization transfer rate, MTRasym]:


$$\text{MTRasym}\left(+3.5\text{ ppm}\right)\;\left(\%\right)\;=\frac{\;\left(S_{-\Delta \omega }-S_{\Delta \omega }\right)}{S_{0}}\times \;100\%$$

where S_0_ is the water signal intensity when the saturation frequency is -1540 ppm, S_-Δω_ and S_Δω_ are the water signal intensities after B0 correction when the saturation frequencys are +3.5 and -3.5 ppm, respectively (16). DWI was quantified by caculation of the ADC map. And the ADC map was obtained by the following mono-exponential model:


S(b) / S(0) = exp(-b · ADC)

where S(0) and S(b) represent the signal intensity for b = 0 and 1000 s/mm^2^, respectively.

Then, the reconstructed DWI and APTWI images are uploaded to the post-processing workstation (Intellispace V10, Philips Healthcare) for subsequent image analysis. Without knowledge of the pathological results, two radiologists with more than 10 years’ experience in musculoskeletal diagnosis independently analyze MTRasym and ADC maps. The region of interest (ROI) for tumor was manually drawn on the MTRasym map with fusion onto the fat-suppressed T2WI image (as reference), and then copied to the ADC map. The criteria for ROI determination were as follows: 1. The ROIs for tumors were drawn at the image slice with the maximum display of the tumor and its adjustent upper and lower slices, and the average value of data measurement from the three slices was used for further analyses. 2. For reduction of the partial volume effect from non-tumor tissues, the ROIs were drawn with 1-2 voxels at the edge of the tumors excluded, and areas including bleeding, necrosis, cystic change, calcification and/or fat/bone morrow contents were also excluded as much as possible.

### 2.4 Statistical analysis

Statistical analysis was performed using SPSS 22.0.0 and MedCalc v.20.0.7 software. The consistency of MTRasym(3.5 ppm) and ADC values measured from ROIs by the two radiologists was evaluated using the intraclass correlation coefficient (ICC), and an ICC>0.75 was considered to indicate good consistency. The mean values by the two observers were taken for subsequent statistical analysis. The normality and homogeneity of variance of data were conformed by the Kolmogorou-Smirnou and Levene tests, respectively. The comparison of each parameter between the two groups was performed using the independent sample t-test. With pathological diagnosis as the standard, the receiver operating characteristic (ROC) curves for APTWI and DWI in the differential diagnosis of benign and malignant bone tumors were calculated. The DeLong test was used to compare the area under the ROC curve (AUC). A P value<0.05 was considered statistically significant.

## 3 Results

### 3.1 General clinical data

**Figure 1:**
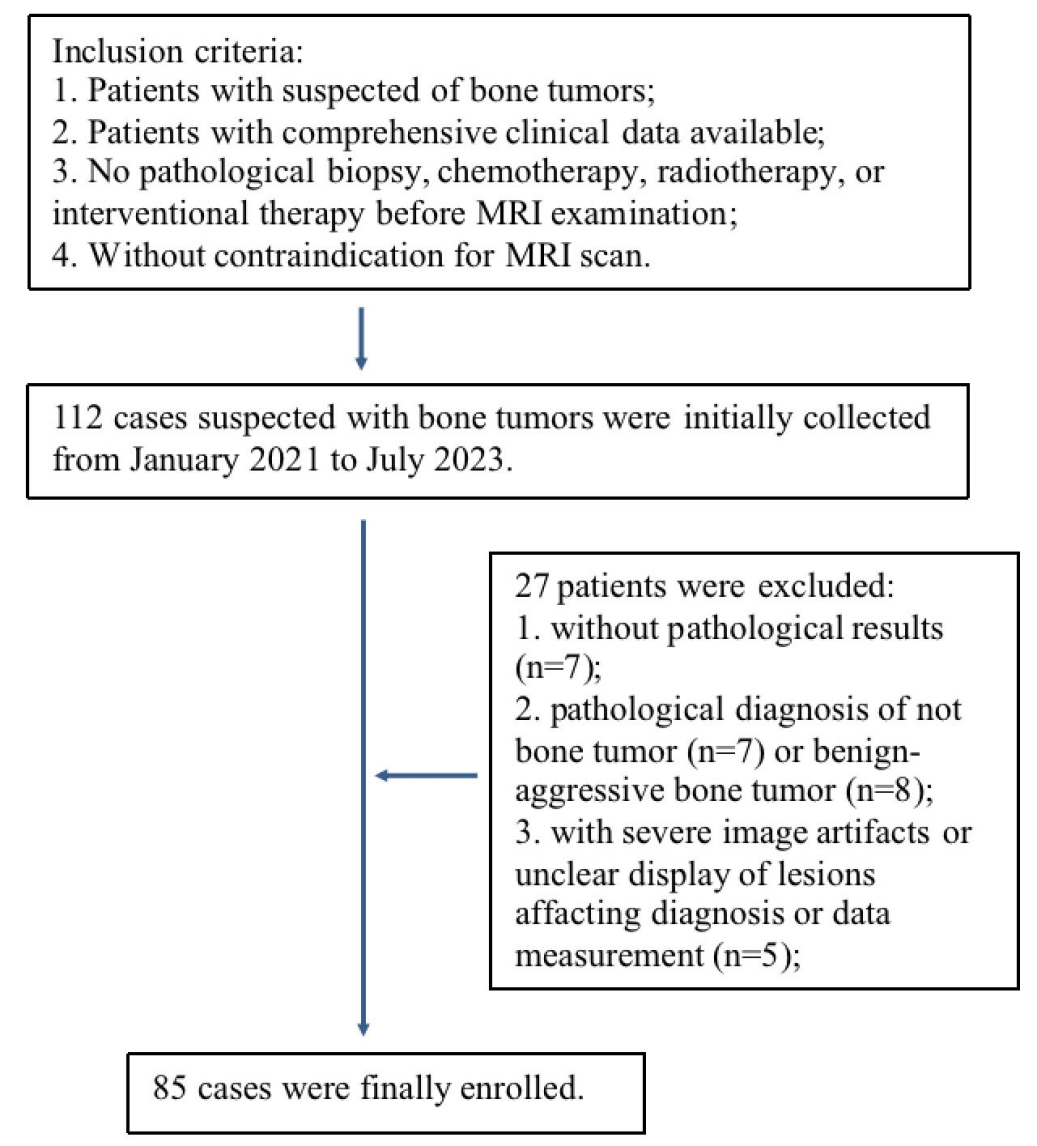
Flowchart of patient selection.

27 of 112 patients were excluded due to the following reasons: 7 patients without pathological results; 7 patients with pathological confirmation of not bone tumors; 8 patients with intermediate tumors that could not be included into benign or malignant goups; and 5 patients with poor image quality due to severe motion artifacts or artifacts by the influence of surrounding fat signals affacting diagnosis and data measurement (**Figure 1**). A total of 85 patients with bone tumors were finally included in the study after patient selection, of which 51 were male and 34 were female, with ages ranging from 7 to 74 years and a median age of 34 years. According to the fifth edition of the WHO Classification of Bone and Soft Tissue Tumors (2020), the bone tumors were classified into 33 benign tumors and 52 malignant tumors (the subtypes for these tumors are provided in **Table 1**).

**Table 1:** The subtype information for all included bone tumors.

Benign (n=33)		Malignant (n=52)	
Fibrous dysplasia	14	Osteosarcoma	28
Enchondroma	6	Chondrosarcoma	10
Non-ossifying fibroma	5	Bone metastases	8
Osteofibrous dysplasia	4	Plasmacytoma of bone	3
Osteochondroma	2	Chordoma	1
Chondroblastoma	1	Lymphoma	1
Chondromyxoid fibroma	1	Parosteal osteosarcoma	1

### 3.2 Differences in APTWI and DWI parameters between benign and malignant bone tumors

**Figure 2:**
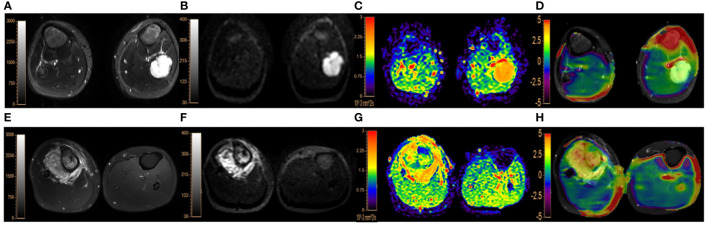
**(A–D)**. A 9-year-old male patient with endochondroma in the left fibula: **(A)**, fat-suppressed T_2_WI showing a irregular hyperintense lesion in the left fibula with marked dilatation, lobulated changes and intact bone cortex; **(B, C)**, DWI image (b=1000 s/mm^2^) and ADC map, both showing the lesion with slightly uneven high signal intensities, with the lesion ADC value of 1.99×10^-3^ mm^2^/s; **(D)**, APTWI fused on fat-suppresed T_2_WI with the lesion showing the MTRasym(3.5 ppm) value of 1.73%. **(E–H)**. a 17-year-old male with osteosarcoma in the right proximal tibia accompanied by surrounding soft tissue mass: **(E)**, fat-suppressed T_2_WI showing mixed high signal and irregular bone destruction in the right proximal tibia with surrounding soft tissue mass; **(F, G)**, DWI image (b=1000 s/mm^2^) showing obvious hyperintensity and ADC map showing hypointensity with the lesion ADC value of 1.18×10^-3^ mm^2^/s; **(H)**, the APTWI image fused on fat-suppresed T_2_WI with the lesion MTRasym(3.5 ppm) value of 4.07%.

The consistency was good for the MTRasym(3.5 ppm) and ADC measurements by the two observers (ICC values were 0.856 and 0.863, respectively). The MTRasym(3.5 ppm) values (2.95 ± 0.63% vs. 2.13 ± 0.89%, P< 0.001) of malignant bone tumors were comparatively higher, while the ADC values (1.22 ± 0.44 vs. 1.71 ± 0.45 ×10^-3^ mm^2^/s, P< 0.001) of malignant bone tumors were comparatively lower than those of benign tumors. There were slight overlaps in the MTRasym(3.5 ppm) and ADC values between benign and malignant bone tumors. (**Table 2**; **Figure 2**).

**Table 2:** Comparison of quantitative APTWI and DWI imaging parameters in patients with benign and malignant bone tumors.

	Malignant (n=52)	Benign (n=33)	*P*
MTRasym(3.5 ppm)(%)	2.95 ± 0.63	2.13 ± 0.89	< 0.001
ADC(×10^-3^ s/mm²)	1.22 ± 0.44	1.71 ± 0.45	<0.001

APTWI, Amide Proton Transfer Weighted Imaging; DWI, Diffusion Weighted Imaging; MTRasym(3.5 ppm), the Asymmetric Magnetization Transfer Rate at an offset of 3.5 ppm; and ADC, the Apparent Diffusion Coefficient.

### 3.3 Diagnostic efficacy of APTWI, DWI, and their combination in differentiation between benign and malignant bone tumors

**Figure 3:**
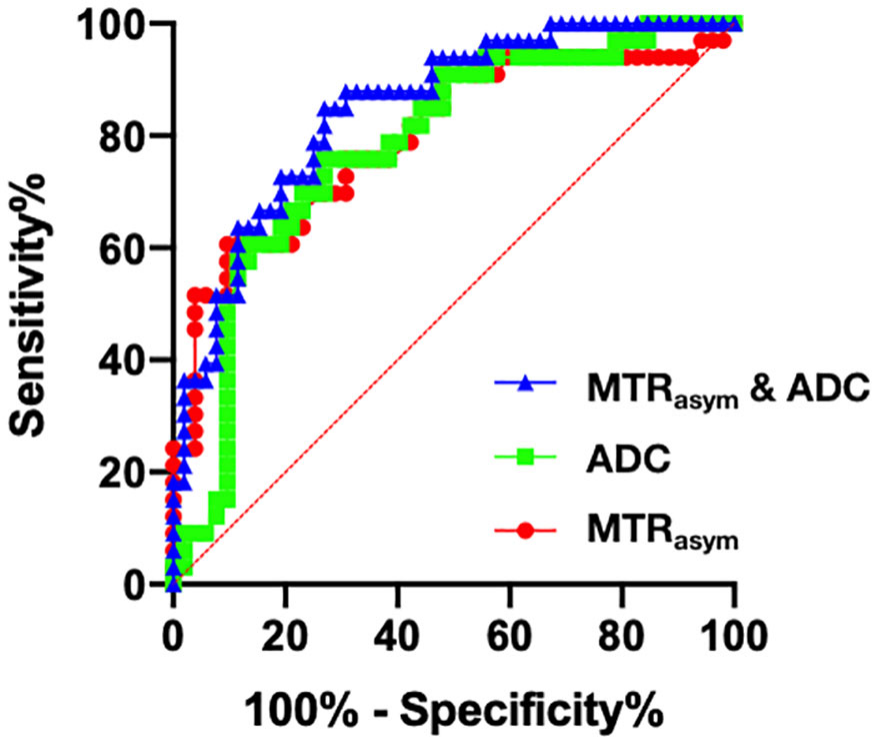
Receiver operating characteristic (ROC) curves of APTWI (MTRasym), DWI (ADC) and their combination for discrimination between benign and malignant bone tumors.

The AUCs of MTRasym(3.5 ppm) and ADC for differential diagnosis between benign and malignant bone tumors were 0.798 and 0.780, respectively. And their combination improve the diagnostic efficacy (AUC = 0.849, sensitivity = 84.9%, and specificity = 73.1%) (**Table 3**; **Figure 3**). There was no significant difference among the AUCs by APTWI, DWI and their combination.

**Table 3:** Diagnostic performance of APTWI, DWI and their combination in differentiation between benign and malignant bone tumors.

	AUC	Sensitivity	Specificity	Cut-off value
MTRasym(3.5 ppm)	0.798	60.6%	90.4%	2.28%
ADC	0.780	75.8%	73.1%	1.34×10^-3^ s/mm²
MTRasym(3.5 ppm) & ADC	0.849	84.8%	73.1%	——

APTWI, Amide Proton Transfer Weighted Imaging; DWI, Diffusion Weighted Imaging; MTRasym(3.5 ppm), the Asymmetric Magnetization Transfer Rate at an offset of 3.5 ppm; and ADC, the Apparent Diffusion Coefficient.

## 4 Discussion

This study investigated the performance of APTWI and its combination with DWI in differentiation between benign and malignant bone tumors. Results indicated that both the MTRasym(3.5 ppm) and ADC values derived from APTWI and DWI, respectively, were comparatively different between benign and malignant bone tumors. The combination of APTWI with DWI helps improve diagnostic efficiency.

Through comparative analysis of 85 bone tumors, this study found that the MTRasym(3.5 ppm) values of malignant tumors were comparatively higher than those of benign tumors, which is consistent with results by previous studies on tumor APTWI (17–22). This indicates that APTWI can help distinguish benign from malignant bone tumors. It is speculated that the malignant tumors are associated with higher proliferation of the tumor cells, and thus increased synthesis of mobile proteins and peptides, as well as an increase in the content of exchangeable amide protons, which can lead to the higher MTRasym(3.5 ppm) values. While different subtypes of benign and malignant bone tumors can be associated with substanal differences in biological characteristics and cellular microenvironment etc, which can also bring variations and thus contribute to the overlap of MTRasym values between benign and malignant bone tumors. Further studies with increased cohort of subjects are needed to take the different subytpes of bone tumors into consideration.

The adipose tissues or fat contents in lesions can bring undesirable artifacts (usually false positive signals) to the APTWI images (23, 24). Actually, very few bone tumors originally contain fat contents. According to the WHO (2020) 5th edition “Classification of Bone and Soft Tissue Tumors” (1), the lipogenic bone tumors have been removed. Benign lipogenic bone tumors in WHO (2013) 4th edition were classified as other mesenchymal bone tumors, while malignant liposarcoma was removed in the new edition. The typical lipomas can also be clearly distinguish from the other type bone tumors by their specific high intensities on fat unsuppressed T1 weighted images. According to the pathological resutls, there was no lipogenic bone tumor included in this study. Therefore, fat contents in the included tumors should be limited. Moreover, area in lesions with suspected containing of fat/bone marrow contents have been excluded as much as possible when drawing the ROIs.

In this study, some bone tumors were associated with cystic changes. And we found that the MTRasym(3.5 ppm) values of the cystic area around the lesion could be significantly higher than those of the solid part of malignant/benign tumors. The cystic component around the tumor may contain more free proteins and peptides. If the cystic area is not avoided when selecting the ROI, it will cause the MTRasym(3.5 ppm) of the tumor to be abnormally high (25). In addition, the previous study has shown that the MTRasym(3.5 ppm) value was positively correlated with the T1 value, and the T1 value of cystic lesions or the cystic part of the lesion relatively long and not easily affected by magnetization transfer (MT) effect, which will further amplify the CEST effect, leading to a high MTRasym(3.5 ppm). In this study, the solid part of the tumor was selected when selecting the ROI during post-processing to avoid the influence on tumor identification caused by the cystic area of the tumor.

Many studies have shown the value of DWI in distinguishing benign and malignant bone tumors (26). The ADC value of malignant tumors is usually lower than that of benign tumors, which is consistent with the results of the current study. In addition, DWI can also be used for grading bone tumors (27), evaluating treatment effects (28), and distinguishing benign and malignant fractures of the spine (29). This study found that the diagnostic efficacy of APWI (AUC = 0.798) in distinguishing benign from malignant bone tumors was slightly higher than DWI (AUC = 0.780) without significant difference. With 2.28% as the optimal cut-off value of MTRasym(3.5 ppm), APTWI can achieve the sensitivity and specificity of 60.6% and 90.4%, respectively. APTWI enables non-invasive evaluation of mobile proteins and peptides, while DWI reflects the microscopic movement of water molecules in tumor tissues, and thus their combination helps further improve the diagnostic efficacy (AUC = 0.849).

This study has some limitations. First, this study simply divided the bone tumors into benign and malignant groups based on pathological results; and 8 cases of intermediate bone tumors, which could not be simply included to benign or malignant groups, were excluded from this study due to the limited size of patient cohort. Further studies with increased sample size are needed to include the different subtypes of bone tumors into consideration and provide more solid statistical results. Second, 5 cases were excluded due to the poor image quality, which may induce potential statistical bias to the remaining cohort. Third, APTWI images were acquired without suppression of signals from fluid compartments; for reducing potential signal contamination, the ROIs were drawn carefully with exclusion of areas such as bleeding, necrosis, and cystic change etc. Further technical development/modifications of APTWI (30) may help improve the clinical diagnostic performance. Fourth, the ROI was manually outlined, which may have certain errors in lesion range. Some more advanced segmentation method may be introduced in further studies for more comprehensive evaluation.

## 5 Conclusion

This study showed that MTRasym(3.5 ppm) values of malignant bone tumors were comparatively higher than those of benign bone tumors, reflecting the abnormal increase of protein synthesis in malignant tumors. APTWI combined with DWI may help improve the clinical differential diagnosis between benign and malignant tumors. Further studies with increased sample size are needed to provide more solid statistical results.

## Data availability statement

The raw data supporting the conclusions of this article will be made available by the authors, without undue reservation.

## Ethics statement

The studies involving humans were approved by Research and Clinical Experiment Ethics Committee of the First Affiliated Hospital of Zhengzhou University. The studies were conducted in accordance with the local legislation and institutional requirements. Written informed consent for participation in this study was provided by the participants’ legal guardians/next of kin. Written informed consent was obtained from the individual(s) for the publication of any potentially identifiable images or data included in this article.

## Author contributions

YL: Writing – original draft, Writing – review & editing, Data curation, Formal Analysis, Project administration, Supervision. LL: Data curation, Formal Analysis, Software, Writing – review & editing. YZ: Conceptualization, Resources, Writing – review & editing. CR: Conceptualization, Resources, Writing – review & editing. WZ: Project administration, Writing – review & editing. JC: Conceptualization, Resources, Supervision, Writing – review & editing.
